# A small viral protein suppresses immune amplification by two distinct mechanisms

**DOI:** 10.1128/mbio.01237-26

**Published:** 2026-07-29

**Authors:** Si Liu, Ting Yang, Qian Cai, Wan-Xiang Li, Shou-Wei Ding

**Affiliations:** 1 National Key Laboratory of Agricultural Microbiology, College of Plant Science and Technology, Hubei Hongshan Laboratory, Huazhong Agricultural University, Wuhan, China; 2 Department of Microbiology & Plant Pathology and Center for Plant Cell Biology, Institute for Integrative Genome Biology, University of California 8790, Riverside, California, USA; Duke University School of Medicine, Durham, North Carolina, USA

**Keywords:** antiviral RNAi, VSR-2b, RDR1, RDR6, dsRNA binding, vsiRNA

## Abstract

Diverse viral suppressors of RNA interference (RNAi) and RNA silencing (VSRs) interact directly with core protein and/or RNA components of the host RNAi pathway. However, the specific counter-defense function of any VSR biochemical activity is fully validated only when it is shown as essential for viral infection in the wild-type but not mutant hosts defective in antiviral RNAi. Here, we investigated the role of VSR activities for direct binding to small-interfering RNA duplexes (siRNA), long double-stranded RNA (dsRNA), or RNA-dependent RNA polymerase 1 (RDR1) during plant infection by wild-type and mutant cucumber mosaic virus (CMV), a positive-strand RNA virus expressing the 110-residue 2b protein as its VSR. We demonstrate that a C-terminally truncated 2b mutant (2b_1-93_) active in direct binding to siRNA and dsRNA, but not RDR1, was able to suppress the amplification of virus-derived siRNAs (vsiRNA) and antiviral RNAi mediated by RDR6, but not RDR1. By contrast, an N-terminally truncated 2b mutant (2b_18-110_) inactive in direct binding to siRNA or dsRNA was able to suppress vsiRNA amplification and antiviral RNAi mediated by RDR1, but not RDR6, and was less effective to promote systemic CMV infection and disease development than 2b_1-93_. Together, our results show that whereas RDR1 suppression requires direct binding of VSR-2b to RDR1, but not siRNA or dsRNA, RDR6 suppression depends on direct binding to siRNA and dsRNA, but not RDR1. Therefore, CMV, through its VSR-2b, suppresses two parallel vsiRNA amplification pathways by distinct molecular mechanisms, and this unique property may account for the unusually wide host range of CMV.

**IMPORTANCE:**

Host amplification of antiviral immunity is essential for robust control of viral infections. However, little is known about the mechanisms that viruses have evolved to suppress immune amplification in plants. Here, we characterized whole plant infection by cucumber mosaic virus (CMV) with its viral suppressor of RNA interference (RNAi) mutated to become inactive in direct binding to small-interfering RNA duplexes (siRNA), long double-stranded RNA (dsRNA), or RNA-dependent RNA polymerase 1 (RDR1). We demonstrate maximal suppression of both RDR1- and RDR6-mediated antiviral RNAi amplification by the CMV 2b protein, a viral suppressor of RNAi (VSR). Notably, whereas RDR1 suppression requires direct binding of 2b to RDR1 but not siRNA or dsRNA, RDR6 suppression depends on direct binding to siRNA and dsRNA, but not RDR1. Our findings reveal a novel counter-defense strategy evolved by a wide host range positive-strand RNA virus to suppress two pathways of immune amplification by distinct mechanisms.

## INTRODUCTION

Viruses infect and cause devastating diseases in plants and animals because viruses have evolved sophisticated strategies to suppress antiviral immune responses and immune amplification. The animal and plant small-interfering RNA (siRNA) pathway of RNA interference (RNAi) is targeted for suppression by a diverse family of viral proteins (1–7), known collectively as viral suppressors of RNAi or RNA silencing (VSR). VSRs are widespread in animal and plant RNA viruses despite their limited coding capacity (6, 8–11). The first VSRs were discovered in the late 1990s by demonstrating their unprecedented activity to suppress RNA silencing/post-transcriptional gene silencing of transgenes in plants, including the helper component-protease (HC-Pro), the 2b protein, and the 19K protein (p19) encoded by potyviruses, cucumoviruses, and tombusviruses, respectively (12–15). Studies on these VSRs as model systems have revealed first insights into the mechanisms of RNAi suppression (5, 6, 8–11, 16–20).

Much is known about the biochemical activities of VSRs characterized frequently in reporter assays (6, 8–11). Many VSRs interact directly with core components of the RNAi pathway, such as siRNA duplexes, long double-stranded RNA (dsRNA), and argonaute proteins (AGOs) (6, 8–11). For example, structural and functional studies have shown that p19 forms a homodimer to selectively sequester siRNA duplexes (1, 2, 5, 18). The 2b protein encoded by cucumber mosaic virus (CMV) and other cucumoviruses ranges from 95 to 112 amino acids in length (21, 22). Despite the small size, the 2b protein of CMV (Cmv2b) has been shown to bind directly to AGO1 and AGO4, siRNA duplexes, and long dsRNA, as well as RNA-dependent RNA polymerase (RdRP) 1 (RDR1) in various assays (22–35). Detailed mutational analyses of Cmv2b, performed mostly on those encoded by subgroup I strains such as Fny-CMV, have mapped specific regions of Cmv2b required for these biochemical activities. The N-terminal half of Cmv2b is sufficient for binding to siRNA duplexes and long dsRNA, with both activities abolished by deleting or specific mutations in the N-terminal 17 amino acid sequence (26, 27, 29–31). The central region of Cmv2b is involved in AGO binding as AGO binding is not disrupted by deleting either the N-terminal 17 or the C-terminal 16 amino acid sequence (26, 35). In contrast, RDR1 binding may involve the entire Cmv2b including the C-terminal 15 amino acid sequence dispensable for binding to AGOs, siRNA duplexes, or long dsRNA (26, 33, 35).

Less is known about the function of the specific VSR biochemical activities during *in vivo* infection of the cognate viruses. In antiviral RNAi, long dsRNA precursors synthesized during viral replication and infection are processed into siRNA duplexes by Dicer or Dicer-like (DCL) nucleases (36–44). These virus-derived small-interfering RNAs (vsiRNAs) are then assembled with an AGO and cofactors into the RNA-induced silencing complex (RISC) to direct specific viral RNA clearance (45–49). Unlike insects and mammals, plants and nematodes encode a family of cellular RdRPs for the amplification of vsiRNAs to enhance the potency of the primary antiviral RNAi response (50–54). Notably, VSR of diverse plant viruses becomes largely dispensable for infection in *RDR1*/*RDR6* double-knockout plants despite the presence of the primary RNAi system for dicing and slicing (51, 55–58). However, little is known about the mechanisms that viruses have evolved to suppress vsiRNA amplification during whole plant infection.

In this work, we investigated the role of the Cmv2b biochemical activities for direct binding to siRNA duplexes, long dsRNA, and RDR1 during CMV infection in the model plant *Arabidopsis thaliana*, a natural host of CMV (59). CMV contains a tripartite positive-strand RNA genome coding for five genes in total, including the 2b gene (59, 60). CMV has a very wide host range, including 1,071 species of plants in 521 genera (61). We characterized whole plant infection by CMV mutants modified to express a mutant Cmv2b protein inactive in direct binding to siRNA duplexes and long dsRNA or RDR1, as defined by previous studies (22–35). Our findings show that Cmv2b directs maximal suppression of vsiRNA amplification by either RDR1 or RDR6 using distinct mechanisms and with different domain requirements. Together, our studies reveal a novel counter-defense strategy evolved by CMV to suppress two parallel pathways of immune amplification by distinct mechanisms, which may play a key role in the virus infecting a wide range of plants.

## RESULTS

### Cmv2b potently suppresses RDR1-mediated antiviral RNAi in a mechanism dependent on direct binding to RDR1

**Fig 1:**
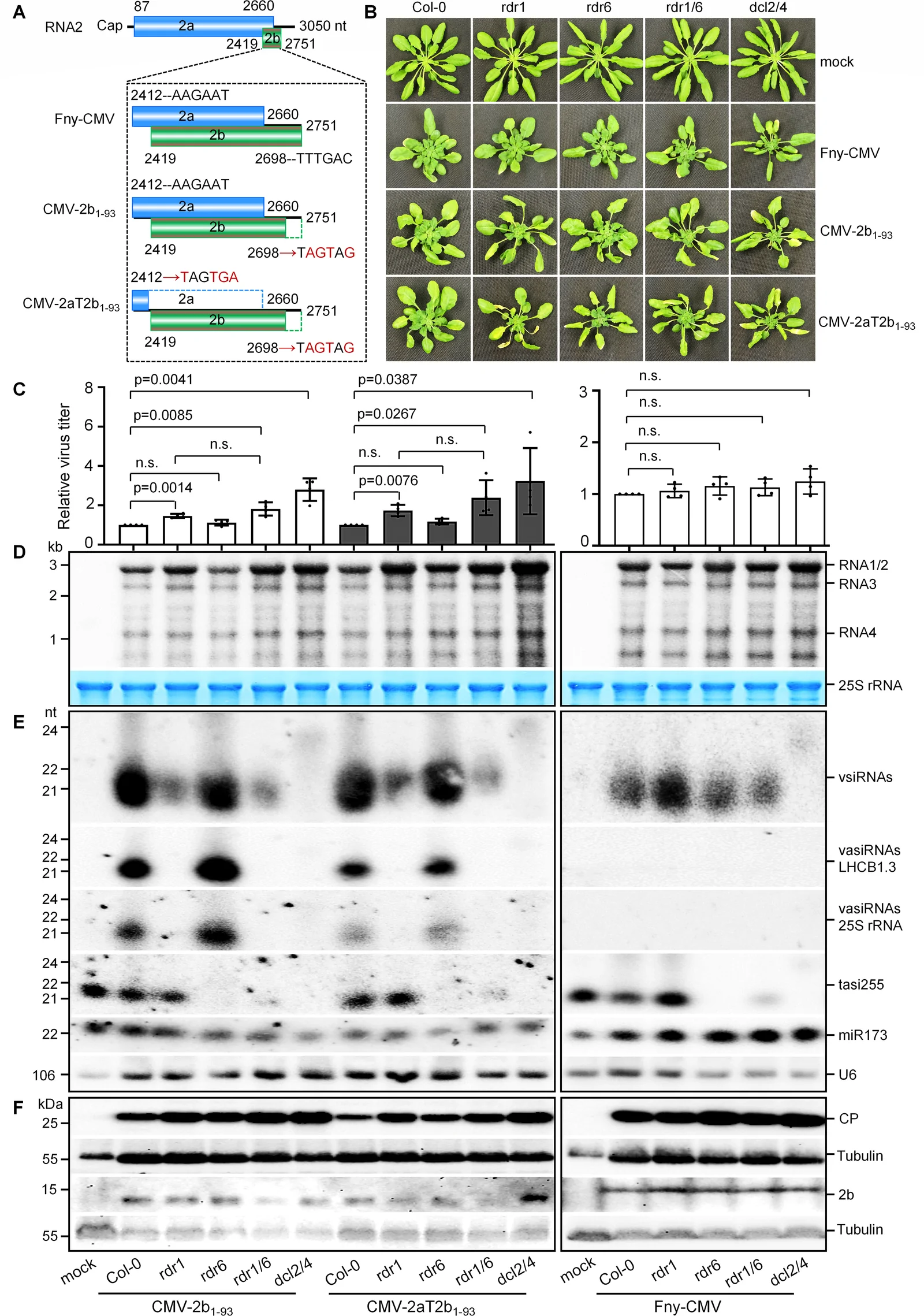
Characterization of whole plant infection by CMV-2aT2b_1-93_, CMV-2b_1-93,_ and Fny-CMV. (**A**) Genome organization of wild-type and mutant Fny-CMV RNA 2 with the first and last nucleotide positions of the overlapping open reading frames (ORF) 2a and 2b indicated. The nucleotide substitutions introduced are marked in red, the C-terminal region truncated in ORFs 2a and 2b indicated, respectively, by blue and green dash line boxes, and the deleted region of RNA represented by a red dash line. (**B**) Wild-type and mutant plants of different genotypes were photographed at 14 days post-inoculation (dpi) with buffer (mock) or purified virions (10 μg/mL) of CMV-2b_1-93_, CMV-2aT2b_1-93,_ or Fny-CMV. (**C–F**) Total RNAs or proteins were extracted from the inoculated plants at 14 dpi and used for Northern blot detection of the viral genomic RNAs (**D**) and of the vsiRNAs, endogenous microRNA-173 (miR173), and trans-acting siRNA-255 (tasi255) as well as the vasiRNAs targeting the *LHCB1.3* mRNA and 25S rRNA (**E**), or Western blot detection of the viral coat protein (CP) and 2b protein (**F**). Staining for 25S rRNA and detection of tubulin alpha chain or U6 RNA on the same membrane were used as loading controls. Virus accumulation levels in the inoculated plants as quantified by the relative Northern blot hybridization signal intensity of the viral RNA 3 with the accumulation level of each virus in Col-0 plants set as 1 (**C**). Data presented are means ± SEM from four independent experiments (the individual values represented by black dots), and *P* values indicate statistical differences of the virus titer between the two indicated genotypes (one-sided unpaired Student’s *t* test with Welch’s correction, n.s., not significant, *P* > 0.05).

We first determined whether the *in vivo* counter-defense function of Cmv2b depends on its biochemical activity for direct binding to RDR1, shown recently between Cmv2b of Fny-CMV and cucumber RDR1 by yeast two-hybrid (Y2H) and bimolecular fluorescence complementation assays (BiFC) (33). Nucleotide substitutions were introduced into the RNA 2 of the Fny-CMV genome to generate a mutant virus, designated CMV-2aT2b_1-93_ (Fig. 1A). The genome of CMV-2aT2b_1-93_ encodes a mutant Cmv2b protein 2b_1-93_ deleted of the C-terminal region essential for direct binding to RDR1 but not siRNA duplexes, long dsRNA, or AGO1 and AGO4 proteins (26, 33, 35). CMV-2aT2b_1-93_ also encodes a C-terminally truncated viral RdRP protein 2a (2aT) as in CMV-2aT∆2b, which exhibits no defects in viral RNA replication in RNAi-defective mutant plants (55, 62–64),

CMV-2aT2b_1-93_ infection was characterized in wild-type (Col-0) and mutant *A. thaliana* plant knockout of one or more genes required for antiviral RNAi, as described previously (65, 66). CMV infection in the absence of Cmv2b expression triggers strong antiviral RNAi directed by 21- and 22-nt vsiRNAs, which are produced, respectively, by DCL4 and DCL2 and amplified further by RDR1 and RDR6 in Col-0 plants (42, 55). We found that unlike CMV-2aT∆2b with severe infection defects described previously (42, 55), CMV-2aT2b_1-93_ was as virulent as wild-type Fny-CMV and induced early development of systemic disease symptoms clearly visible at 14 days post-inoculation (dpi) not only in all mutant plants but also in Col-0 plants (Fig. 1B). Compared to Col-0 plants, however, CMV-2aT2b_1-93_ replicated to significantly higher levels in both *rdr1 rdr6* and *dcl2 dcl4* mutant plants, as revealed by the detection of the viral coat protein (CP) and genomic RNAs (Fig. 1C, D, and F, left panel). As shown previously (67), Fny-CMV accumulated to similar levels in the infected Col-0, *rdr1 rdr6,* and *dcl2 dcl4* plants (Fig. 1C, D, and F, right panel). These results demonstrated the inhibition of CMV-2aT2b_1-93_ accumulation in Col-0 plants by an antiviral RNAi pathway dependent on *RDR1*, *RDR6,* or both.

Northern and Western blotting further showed that CMV-2aT2b_1-93_ also replicated to high levels in *rdr1* single-mutant plants as in *rdr1 rdr6* plants, whereas the mutant virus titers in *rdr6* plants remained as low as in Col-0 plants (Fig. 1C, D, and F, left panel). Notably, small RNA blotting revealed that despite higher viral replication levels in *rdr1* and *rdr1 rdr6* plants, these mutant plants produced markedly lower levels of vsiRNAs than Col-0 and *rdr6* plants (Fig. 1E, left panel). However, no significant differences in the accumulation levels of the viral genomic RNAs or CP were observed among Col-0, *rdr1*, *rdr6,* and *rdr1 rdr6* plants infected with Fny-CMV, and the genetic knockout of *RDR1* and/or *RDR6* did not lead to reduced accumulation of the vsiRNAs (Fig. 1C through E, right panel). These findings revealed active RDR1-dependent vsiRNA amplification and antiviral RNAi during infection with CMV-2aT2b_1-93,_ but not Fny-CMV.

There is no detectable vsiRNA amplification in plants infected with Fny-CMV that directs the expression of wild-type Cmv2b protein (30, 67). Thus, RDR1-dependent vsiRNA amplification and antiviral RNAi detected during CMV-2aT2b_1-93_ infection illustrate that, unlike the full-length Cmv2b (Fig. 1C through E, right panel), vsiRNA amplification by RDR1 is not suppressed by the mutant Cmv2b deleted of the C-terminal region essential for direct binding to RDR1. Together, our results demonstrate maximal suppression of RDR1-dependent immune amplification by Cmv2b in a mechanism dependent on direct binding to RDR1.

Wild-type Cmv2b of Fny-CMV potently suppresses RDR1-dependent biogenesis of the virus-activated siRNAs (vasiRNAs) in the infected Col-0 plants (68). We found that, unlike Fny-CMV infection (68), two types of vasiRNAs accumulated to high levels in both Col-0 and *rdr6* plants after CMV-2aT2b_1-93_ infection (Fig. 1E, left panel). Consistently, these RDR1-dependent vasiRNAs remained undetectable in the *rdr1* and *rdr1 rdr6* plants infected with CMV-2aT2b_1-93_ (Fig. 1E, left panel), like in all the wild-type and mutant plants infected with Fny-CMV (Fig. 1E, right panel). These findings provide another line of evidence for the indispensable role of direct binding to RDR1 in the suppression of RDR1-dependent siRNA biogenesis by Cmv2b.

To investigate whether the C-terminal truncation of the viral 2a protein played a role in the novel phenotypes of CMV-2aT2b_1-93_, we generated another Fny-CMV mutant, designated CMV-2b_1-93_ (Fig. 1A). CMV-2b_1-93_ encodes the full-length 2a protein and contains only the substitutions to introduce the C-terminal truncation of Cmv2b as in CMV-2aT2b_1-93_ (Fig. 1A). We detected no obvious differences in the symptom severity of the same panel of wild-type and mutant plants infected with either CMV-2b_1-93_ or CMV-2aT2b_1-93_ (Fig. 1B). Like CMV-2aT2b_1-93_, CMV-2b_1-93_ also replicated to higher levels, and its vsiRNAs accumulated to lower levels in *rdr1* and *rdr1 rdr6* plants than in Col-0 or *rdr6* plants (Fig. 1C through F). The mutant Cmv2b_1-93_ protein was detectable in the plants infected with either CMV-2b_1-93_ or CMV-2aT2b_1-93_ (Fig. 1F) by the polyclonal antisera raised against the recombinant full-length 2b fused with a 6His tag (23). Unlike the full-length 2b protein, however, the accumulation level of Cmv2b_1-93,_ as detected in the Western blotting by the antisera, did not always correlate with those of the viral genomic RNAs and CP for unknown reason(s). Moreover, the vasiRNAs were abundant in Col-0 and *rdr6* plants but undetectable in *rdr1* and *rdr1 rdr6,* as well as *dcl2 dcl4* plants after infection with either CMV-2aT2b_1-93_ or CMV-2b_1-93_ (Fig. 1E). These findings further confirm an essential role for direct binding to RDR1 in suppression of the RDR1-dependent antiviral RNAi responses by Cmv2b.

**Fig 2:**
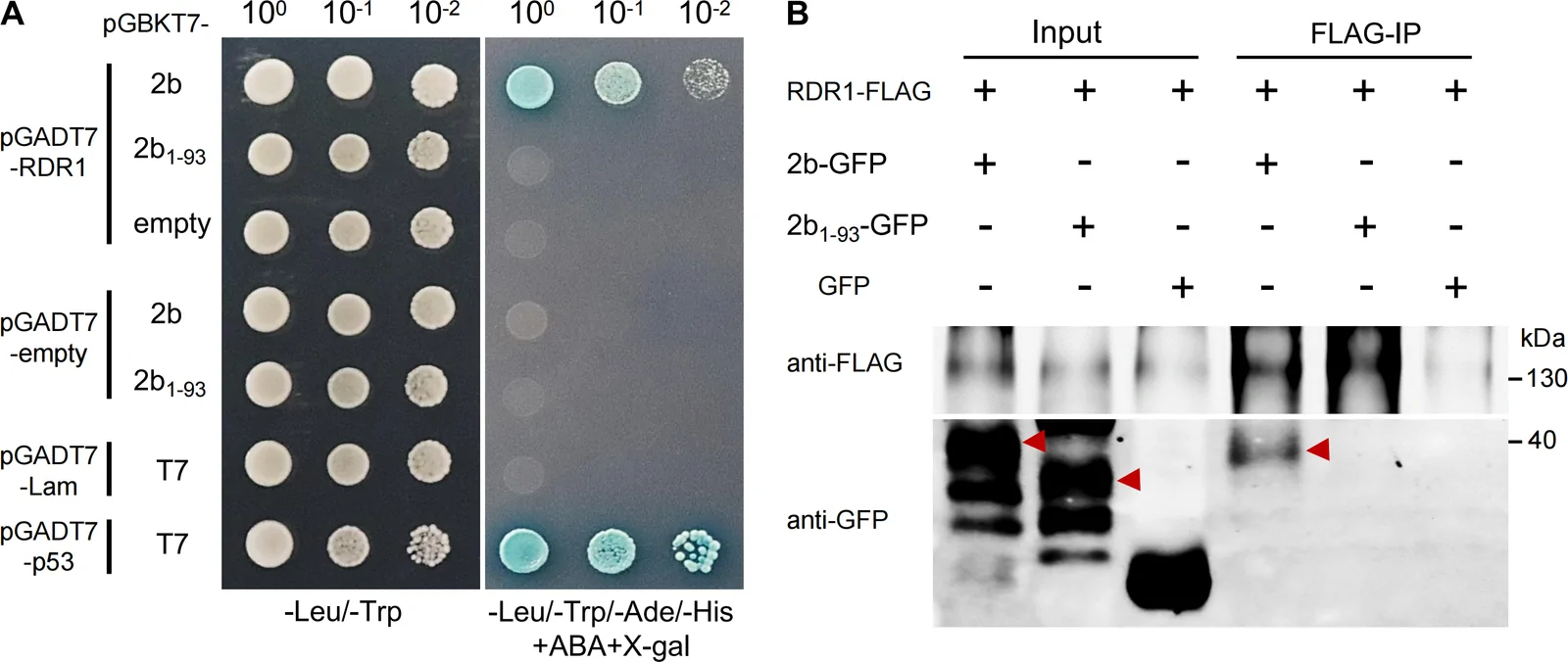
Characterization of the interaction of Cmv2b with *Arabidopsis* RDR1 protein. (**A**) Detect the interaction of *Arabidopsis* RDR1 protein in yeast two-hybrid assay (Y2H) with Cmv2b, but not 2b_1-93_. Yeast cells co-transformed with plasmids as indicated were 10-fold serially diluted and cultured on the double or quadruple amino acid-dropout selection medium plates. Yeast cells co-transformed with pGBK-T7/pGAD-p53 or pGBK-T7/pGAD-Lam were used as positive and negative controls, respectively. Photographs were taken at 3 days post-incubation at 30°C. (**B**) *In vivo* interaction of *Arabidopsis* RDR1 protein with Cmv2b, but not 2b_1-93_. *Arabidopsis* RDR1-FLAG was transiently co-expressed with Cmv2b-GFP, 2b_1-93_-GFP, or GFP in *N. benthamiana* leaves. At 48 h post-*Agrobacterium* infiltration, total proteins were extracted and used for co-immunoprecipitation (IP) with the FLAG-specific antibody. The total protein before and after IP labeled as input and IP, respectively, were analyzed by Western blot using anti-FLAG or anti-GFP specific antibodies.

We found that, like the RDR1 of cucumber plants (33), *Arabidopsis* RDR1 interacted with the full-length Cmv2b protein in Y2H assay (Fig. 2A), which rescued the yeast growth on the quadruple-dropout selection medium as the positive control. However, we detected no interaction between *Arabidopsis* RDR1 and the mutant 2b_1-93_ protein in the same assay (Fig. 2A). To examine whether there is an interaction between Cmv2b and RDR1 *in vivo*, we transiently expressed *Arabidopsis* RDR1 together with either Cmv2b or 2b_1-93_ protein in *Nicotiana benthamiana*. Fig. 2B shows that the antibody to RDR1-flag specifically coimmunoprecipitated the full-length Cmv2b fused with GFP (2b-GFP), but not 2b_1-93_-GFP or GFP, indicating that the C-terminal region of Cmv2b is essential for the RDR1-Cmv2b interaction *in vivo*. Together, our findings demonstrate that, unlike the full-length Cmv2b, the mutant 2b_1-93_ protein is inactive to either interact with RDR1 or suppress RDR1-dependent antiviral RNAi.

### Cmv2b potently suppresses RDR6-mediated antiviral RNAi in a mechanism dependent on direct binding to siRNA and dsRNA, but not RDR1

**Fig 3:**
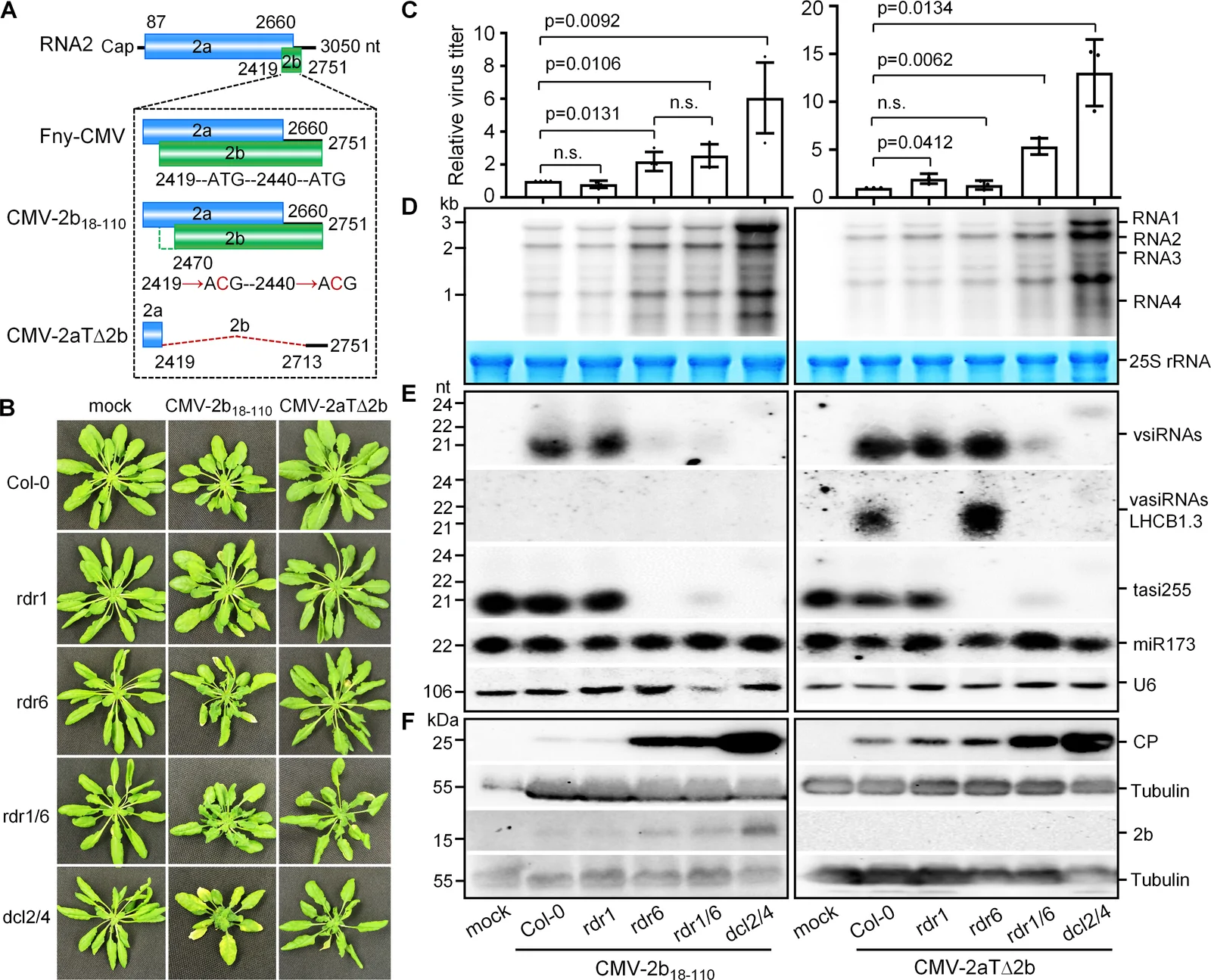
Characterization of whole plant infection by CMV-2b_18-110_. (**A**) Genome organization of wild-type and mutant Fny-CMV RNA 2. The nucleotide substitutions introduced are marked in red, the N-terminal region truncated in ORF2b is indicated by the green dash line box, and the deleted region of the viral genomic RNA 2 is represented by a red dash line. (**B**) Wild-type and mutant plants of different genotypes were photographed at 14 days post-inoculation (dpi) with buffer (mock) or purified virions (10 μg/mL) of CMV-2b_18-110_. (**C–F**) Virus accumulation levels in the inoculated plants at 14 dpi quantified by the relative Northern blot hybridization signal intensity of the viral RNA 3 (**C**), Northern blot detection of the viral genomic RNAs (**D**), and the vsiRNAs and vasiRNAs targeting the *LHCB1.3* mRNA and 25S rRNA as well as endogenous miR173 and tasi255. (**E**) Western blot detection of the viral coat protein (CP) or 2b protein (**F**) in the inoculated plants at 14 dpi, as described in the legend to Fig. 1. Data presented in panel **C** are means ± SEM from four independent experiments (the individual values represented by black dots), and *P* values indicate statistical differences of the virus titer between the two indicated genotypes (one-sided unpaired Student’s *t* test with Welch’s correction, n.s., not significant, *P* > 0.05).

We next determined whether the *in vivo* counter-defense function of Cmv2b requires its biochemical activity for direct binding to siRNA duplexes and long dsRNA shown by electrophoretic mobility shift assays (EMSA) (26, 27, 29–31). To this end, we generated CMV-2b_18-110_ (Fig. 3A), which differs from Fny-CMV only by the deletion of the N-terminal 17 amino acid sequence of Cmv2b essential for direct binding to siRNA and long dsRNA, but not AGO1 and AGO4 proteins (30, 31, 35). We found that CMV-2b_18-110_ established systemic infection in all the wild-type and mutant plants inoculated but induced disease symptoms clearly visible by 14 dpi only in *rdr6*, *rdr1 rdr6* and *dcl2 dcl4* mutant plants (Fig. 3B and D, left panel). CMV-2b_18-110_ titers were higher in *rdr1 rdr6* and *dcl2 dcl4* plants than Col-0 plants, demonstrating active antiviral RNAi against CMV-2b_18-110_ infection. Moreover, CMV-2b_18-110_ replicated to higher levels, and its vsiRNAs accumulated to lower levels in *rdr6* and *rdr1 rdr6* plants than Col-0 or *rdr1* plants (Fig. 3C through F, left panels), indicating that antiviral RNAi against CMV-2b_18-110_ was directed by a pathway dependent on vsiRNA amplification by RDR6, but not RDR1. These findings show that in addition to suppressing RDR1-mediated antiviral RNAi shown above, Cmv2b also suppresses the RDR6-dependent vsiRNA amplification and antiviral RNAi by a mechanism dependent on direct binding to siRNA duplex and long dsRNA.

As shown in Fig. 1, however, the antiviral activity of RDR6 was not detectable in Col-0 plants infected with either CMV-2b_1-93_ or CMV-2aT2b_1-93_. Like Fny-CMV infection (30, 67), each of the two mutant viruses replicated to similar levels either between Col-0 and *rdr6* plants or between *rdr1* and *rdr1 rdr6* plants (Fig. 1C, D, and F), and *RDR6* knockout in the Col-0 or *rdr1* background did not decrease vsiRNA accumulation (Fig. 1E). These results showed that direct binding to RDR1 is dispensable for the maximal suppression of the RDR6-mediated antiviral RNAi by Cmv2b.

We further noted that CMV-2b_18-110_ replicated to similar levels either between Col-0 and *rdr1* plants or between *rdr6* and *rdr1 rdr6* plants (Fig. 3C, D, and F, left panels) and that *RDR1* knockout in the Col-0 or *rdr6* background was not associated with decreased accumulation of the vsiRNAs (Fig. 3E, left panel). These findings demonstrated maximal suppression of the RDR1-mediated antiviral RNAi during CMV-2b_18-110_ infection. Consistently, the vasiRNAs were below the limit of detection in all the wild-type and mutant plants infected with CMV-2b_18-110_ (Fig. 3E, left panel), like Fny-CMV infection (30, 67). Therefore, our results showed that direct binding to siRNA and long dsRNA is dispensable for Cmv2b to suppress the RDR1-mediated antiviral RNAi.

We have shown previously that vsiRNA amplification by either RDR1 or RDR6 confers potent antiviral RNAi against CMV-2aT∆2b, a mutant CMV with the entire 2b coding sequence deleted (55) (Fig. 3A). Consistent with the previous studies (55, 65), we found that unlike CMV-2aT2b_1-93_ or CMV-2b_18-110_, CMV-2aT∆2b accumulated to significantly lower levels and triggered more abundant production of vsiRNAs in both *rdr1* and *rdr6* single-mutant plants than *rdr1 rdr6* double-mutant plants (Fig. 3C through E, right panels). Thus, vsiRNA amplification by either RDR1 or RDR6 is sufficient to facilitate strong antiviral RNAi against CMV in the absence of the viral Cmv2b to suppress vsiRNA amplification by direct binding to RDR1 or siRNA and dsRNA.

### Maximal RDR6 suppression promotes more virulent infection than maximal RDR1 suppression

Our above-described findings reveal distinct mechanisms in the maximal suppression of two vsiRNA amplification pathways by Cmv2b. Interestingly, the mutant Cmv2b proteins encoded by CMV-2b_1-93_ and CMV-2b_18-110_ exhibit opposite VSR activities. Whereas 2b_18-110_ suppresses antiviral RNAi mediated by RDR1 but not RDR6, 2b_1-93_ inhibits antiviral RNAi mediated by RDR6, but not RDR1. Next, we assessed the relative functional significance of the two distinct VSR activities during *in vivo* infection by comparing the infection of wild-type Col-0 plants after mechanical inoculation with equal amounts of CMV-2b_1-93_ and CMV-2b_18-110_ virions.

**Fig 4:**
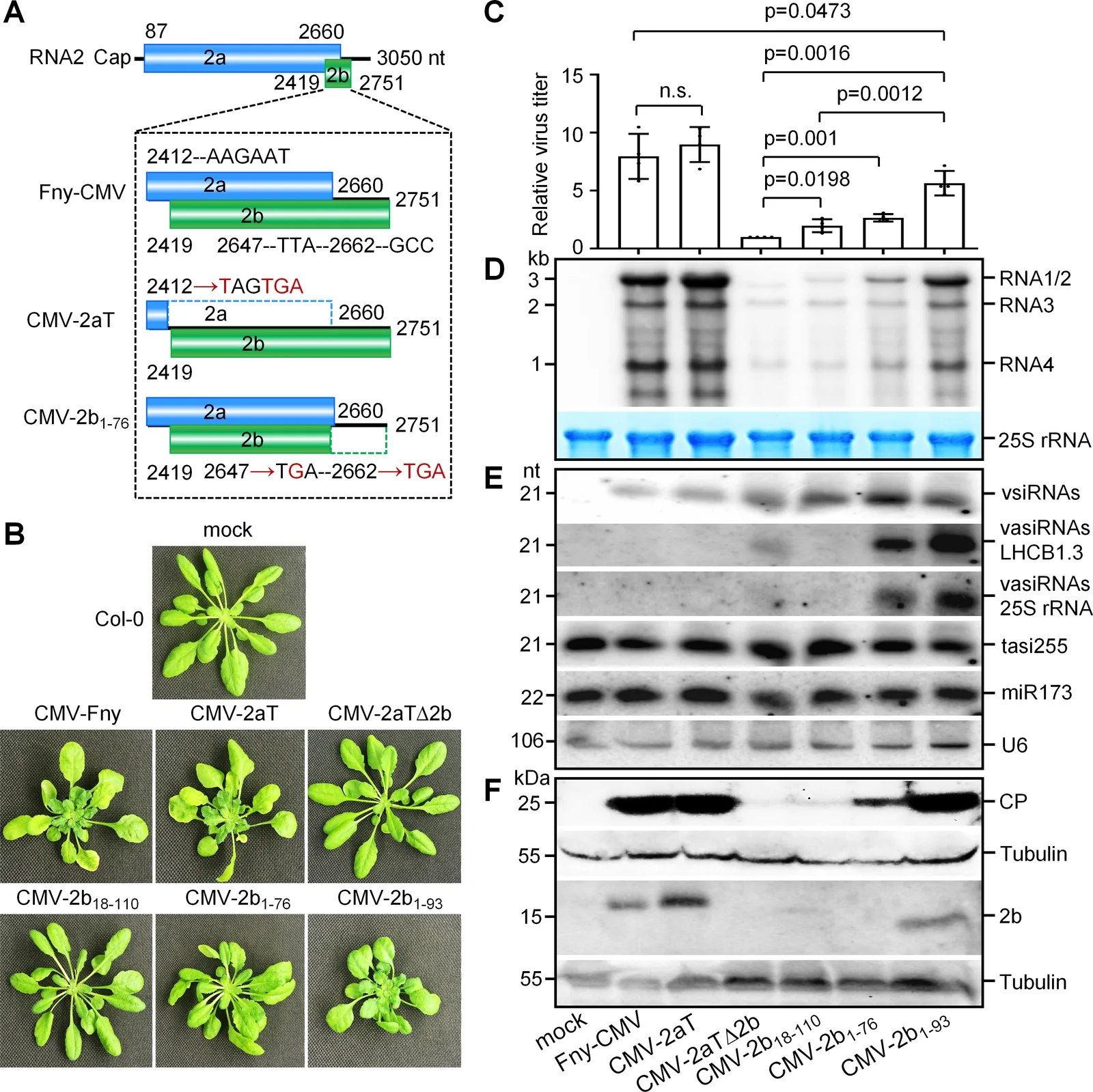
Comparing Col-0 plant infection by wild-type and mutant viruses. (**A**) Genome organization of wild-type and mutant Fny-CMV RNA 2. The nucleotide substitutions introduced are marked in red, and the C-terminal region truncated in ORFs 2a and 2b is indicated, respectively, by blue and green dash line boxes. (**B**) Col-0 plants were photographed at 14 days post-inoculation (dpi) with buffer (mock) or purified virions (10 μg/mL) of wild-type and mutant viruses, as indicated. (**C–F**) Virus accumulation levels in the inoculated plants at 14 dpi, quantified by the relative Northern blot hybridization signal intensity of the viral RNA 3 (**C**), Northern blot detection of the viral genomic RNAs (**D**) and the vsiRNAs and the vasiRNAs targeting the *LHCB1.3* mRNA and 25S rRNA as well as endogenous miR173 and tasi255 (**E**), and Western blot detection of the viral coat protein (CP) or 2b protein (**F**) in the inoculated plants at 14 dpi, as described in the legend to Fig. 1. Data presented in panel **C** are means ± SEM from four independent experiments (the individual values represented by black dots), and *P* values indicate statistical differences in the virus titer between the two indicated genotypes (one-sided unpaired Student’s *t* test with Welch’s correction, n.s., not significant, *P* > 0.05).

We noted the apparent different molecular weights of the wild-type and mutant Cmv2b proteins encoded by Fny-CMV, CMV-2b_1-93,_ and CMV-2b_18-110_ in the Western blot analysis of total proteins extracted from the infected plants (Fig. 4F). Consistent with partial suppression of antiviral RNAi, both CMV-2b_1-93_ and CMV-2b_18-110_ replicated to higher and lower levels, respectively, than CMV-2aT∆2b and Fny-CMV (Fig. 4D and F), suggesting additive effects of RDR1 and RDR6 suppression by Cmv2b. Also, as expected, the vsiRNAs accumulated to markedly higher levels in Col-0 plants infected with either mutant virus than Fny-CMV (Fig. 4C through E). No obvious differences were detected in the accumulation of host microRNA 173 (miR173) or an RDR6-dependent host trans-acting siRNA (tasi255) between the plants with or without infection by wild-type and mutant CMV (Fig. 4E). However, the RDR1-dependent host vasiRNAs were abundant in the CMV-2b_1-93_-infected plants but were undetectable in the mock-inoculated plants or those plants after infection by either Fny-CMV or CMV-2b_18-110_ (Fig. 4E). Like wild-type Cmv2b protein (67, 68), therefore, RDR1-mediated biogenesis of vasiRNAs is suppressed by 2b_18-110,_ but not 2b_1-93_.

We found that CMV-2b_1-93_ accumulated to much higher levels than CMV-2b_18-110_ in the infected Col-0 plants (Fig. 4C, D, and F). Moreover, like Fny-CMV, CMV-2b_1-93_ induced early development of disease symptoms, whereas Col-0 plants infected with CMV-2b_18-110_ developed no obvious disease symptoms at 14 dpi, like CMV-2aT∆2b (Fig. 4B). These results show that maximal suppression of RDR6-dependent antiviral activity is more effective in promoting *in vivo* infection and virulence than maximal suppression of the parallel RDR1-dependent antiviral RNAi.

As further controls, we constructed two additional Fny-CMV mutants, CMV-2aT and CMV-2b_1-76_ (Fig. 4A). CMV-2aT encodes the wild-type Cmv2b protein but directs expression of the same truncated 2a protein as in CMV-2aT2b_1-93_. Like Fny-CMV, CMV-2aT induced severe disease symptoms and replicated to high levels in Col-0 plants, which produced only low levels of vsiRNAs (Fig. 4B through F). Moreover, the vasiRNAs were undetectable in Col-0 plants infected with CMV-2aT, like Fny-CMV and CMV-2b_18-110_ (Fig. 1E, 3E, and 4E), confirming that the C-terminal region of the viral 2a protein is not involved in the suppression of RDR1-dependent vasiRNA production by Cmv2b.

CMV-2b_1-76_ contains a longer C-terminal truncation of Cmv2b than CMV-2b_1-93_ (Fig. 4A). We found that, in infected Col-0 plants, CMV-2b_1-76_ did not induce early development of disease symptoms and accumulated to levels lower than CMV-2b_1-93_ but higher than CMV-2b_18-110_ or CMV-2aT∆2b (Fig. 4B through D and F). Like CMV-2b_1-93_, CMV-2b_1-76_ infection triggered the production of abundant vasiRNAs (Fig. 4E), indicating that the longer C-terminal deletion also abolished the activity of Cmv2b to suppress the RDR1-dependent biogenesis of vasiRNAs.

## DISCUSSION

Little is known about how viruses suppress the amplification of vsiRNAs to promote whole-plant infection. Here, we discovered a novel counter-defense strategy of CMV to suppress two pathways of vsiRNA amplification by the same Cmv2b protein. Notably, despite its small size, Cmv2b employs distinct mechanisms to block the parallel antiviral RNAi pathways.

Our findings provide the first mechanistic insights into the viral suppression of RDR1-dependent antiviral RNAi. Previous studies indicated efficient suppression of RDR1-dependent antiviral responses by Cmv2b because RDR1-mediated amplification of vsiRNAs and vasiRNAs becomes undetectable in plants infected with CMV directing Cmv2b expression (42, 55, 67, 68). Recent work showed that Cmv2b of Fny-CMV, but not 2b_1-93_ protein, directly interacts with both the RNA recognition motif (RRM) and RdRP domains of cucumber RDR1 (33). Here, we found that, unlike wild-type Fny-CMV, CMV-2b_1-93_ infection triggered efficient RDR1-dependent vsiRNA amplification and antiviral RNAi, as well as vasiRNA biogenesis (Fig. 1 and 4), despite the expression of 2b_1-93_, which is sufficient to direct maximal suppression of RDR6-dependent vsiRNA amplification and antiviral RNAi (Fig. 1). As the C-terminal region of Cmv2b deleted in the mutant 2b_1-93_ protein is indispensable for direct binding to RDR1 of both cucumber (33) and *Arabidopsis* plants (Fig. 2) and the infection defects of CMV-2b_1-93_ were efficiently rescued in *rdr1* mutant plants (Fig. 1), our results show that direct interaction with RDR1 is essential for suppressing RDR1-dependent amplification of antiviral RNAi by Cmv2b. In contrast, the RDR1-mediated antiviral responses were all suppressed at the highest level by 2b_18-110_ since *RDR1* knockout in Col-0 and *rdr6* backgrounds neither enhanced virus accumulation nor reduced the accumulation of vsiRNAs and vasiRNAs (Fig. 3 and 4). It is known that 2b_18-110_ is defective in direct binding to both long dsRNA and siRNA duplexes (26, 27, 29–31). Thus, our findings further show that the suppression of RDR1-dependent biogenesis of vsiRNAs and vasiRNAs by Cmv2b is independent of its activity for direct binding to either siRNA duplexes or long dsRNA.

Our results revealed that RDR6-dependent vsiRNA amplification and antiviral RNAi were suppressed at the highest level during CMV-2b_1-93_ infection (Fig. 1 and 4). These results indicate that direct interaction with RDR1 is not necessary for the maximal suppression of RDR6-dependent antiviral RNAi by Cmv2b. In contrast, *RDR6* knockout reduced the vsiRNA levels and enhanced virus titers in plants infected with CMV-2b_18-110_ (Fig. 3 and 4), indicating that direct binding to siRNA and long dsRNA is essential for suppressing RDR6-dependent antiviral RNAi by Cmv2b. Our results are consistent with the characterization of Fny-CMV mutants carrying alanine substitution of the 15th and/or 18th amino acid of the Cmv2b protein and the Cmv2b deletion analysis in the SD strain of CMV in earlier studies (30, 31). In transgenic plants, transgene mRNA bound to the 22-nt siRNA-AGO1 complex is often converted to long dsRNA by RDR6, followed by the dicing of the long dsRNA into secondary siRNAs to sustain transgene RNA silencing (69). Thus, Cmv2b interaction with siRNA duplexes and long dsRNA may inhibit the assembly of siRNA-AGO1 complex and/or the subsequent dicing of long dsRNA in RDR6-dependent antiviral RNAi.

In summary, our work demonstrates that whereas the RDR1-binding activity of Cmv2b functions to promote CMV infection in wild-type and *rdr6* mutant plants, but not *rdr1* mutant plants, the siRNA- and dsRNA-sequestering activity of Cmv2b enhances CMV infection in wild-type and *rdr1* mutant plants, but not *rdr6* mutant plants. These genetic studies illustrate dual suppression of RDR1- and RDR6-mediated vsiRNA amplification and antiviral RNAi by Cmv2b via distinct biochemical mechanisms. Although no additional VSR is known to target RDR1, several VSRs have been shown to suppress RDR6-dependent biogenesis of secondary siRNAs in transgene RNA silencing assays (70–76). However, genetic mutant plants and/or viruses defective, respectively, in the induction and suppression of vsiRNA amplification are not available to establish whether these VSRs promote their cognate viral infection by specifically interfering with RDR6-mediated vsiRNA amplification and antiviral RNAi. A recent study illustrates that the P3 protein of rice grassy stunt virus (RGSV) acts to promote viral infection and virulence by blocking the strigolactone hormone signaling-dependent transcriptional activation of RDR1 and RDR6 genes via sequestering the hormone receptor D14. Accordingly, single-residue substitution in D14 that eliminates its interaction with P3 without affecting hormone perception confers RGSV resistance in two genome-edited rice cultivars (77). It is of interest to note that the Cmv2b is encoded by an overlapping gene evolved more recently than the remaining viral genes (60). We propose that targeting more than one immune pathway by Cmv2b plays a role in the unusually wide host range of CMVs by preventing host plants from easily evolving virus resistance.

## MATERIALS AND METHODS

### Plasmids and viruses

The infectious cDNA clones for wild-type Fny-CMV RNAs 1–3 (pFny109, pFny209, and pFny309) and RNA 2 mutants of CMV-2aT∆2b and CMV-∆2b were described previously (55, 62–64). For the construction of the five RNA 2 mutants in this work, the *Eco*RI-*Pst*I fragment (corresponding to nucleotides 2,133 to 3,050 of RNA2) from pFny209 or CMV-∆2b RNA 2 cDNA clone was first cloned into pGEM-3Z, producing pGEM-2b and pGEM-∆2b, respectively. The RNA 2 mutations were introduced into the *Eco*RI-*Pst*I insert in pGEM-2b or pGEM-∆2b by using the QuickChange Site-Directed Mutagenesis kit 200519 (Agilent Technologies, CA) according to the manufacturer’s instructions and primer pairs described in Table S1. pGEM-2b was used for the construction of all the RNA 2 mutants except pFny209-2b_18-110_, which was based on pGEM-∆2b.

pGEM-2b_1-93_ contains four nucleotide substitutions that change the sequence 2698-TTTGAC-2703 of RNA 2 to TAGTAG (the substituted nucleotides are underlined), converting the 94th and 95th codons of ORF2b to tandem stop codons. Two tandem stop codons were introduced into ORF2a by changing the sequence 2412-AAGAAT-2417 of RNA 2 to TAGTGA, yielding pGEM-2aT. pGEM-2aT2b_1-93_ contains both sets of nucleotide substitutions introduced, respectively, in pGEM-2b_1-93_ and pGEM-2aT, resulting in early termination of both ORFs 2a and 2b by two tandem stop codons. pGEM-2b_1-76_ contains four nucleotide substitutions that convert the 77th and 81st codons of ORF2b into stop codons. pGEM-2b_18-110_ contains two nucleotide substitutions that convert the 1st and 8th ATG codons of ORF2b to ACG so that the 18th codon ATG serves as the start codon of the mutant ORF2b. After verification of the introduced mutations by DNA sequencing, the *Eco*RI-*Pst*I fragment in pGEM-2b_1-93_, pGEM-2aT, pGEM-2aT2b_1-93_, pGEM-2b_1-76,_ and pGEM-2b_18-110_ was cloned back into pFny209, yielding pFny209-2b_1-93_, pFny209-2aT, pFny209-2aT2b_1-93_, pFny209-2b_1-76,_ and pFny209-2b_18-110_, respectively.

Mutant viruses CMV-2b_1-93_, CMV-2aT, CMV-2aT2b_1-93_, CMV-2b_1-76,_ and CMV-2b_18-110_ were propagated and purified from *Nicotiana clevelandii* plants inoculated with *in vitro* RNA transcripts of pFny109 and pFny309 combined with those from pFny209-2b_1-93_, pFny209-2aT, pFny209-2aT2b_1-93_, pFny209-2b,_1-76_ or pFny209-2b_18-110,_ as described (42, 62, 64). The mutations introduced into RNA 2 of each mutant virus and the absence of additional mutations were verified by DNA sequencing of the cDNA fragments amplified by RT-PCR using purified virion RNAs as the template and primer pairs CMV-RNA2Fp and CMV-RNA2Rp (Table S1).

### Plant materials and virus infection assays

*A. thaliana* single- and double-mutant plants *rdr1-1*, *rdr6-15*, *rdr1-1 rdr6-15*, and *dcl2-1 dcl4-2* were described previously (39–42, 55, 63, 78–81). All mutant plants used were in the wild-type Col-0 background. Seeds were stratified at 4°C in dark for 3 days and grown at a greenhouse with 10-h light to 14-h dark cycle at 23°C. Purified virions (10 μg/mL) of wild-type and mutant Fny-CMV viruses were used to mechanically inoculate to 5-week-old *A. thaliana* seedlings. DNA sequencing of the cDNA fragments amplified by RT-PCR using total RNAs as the template extracted from upper non-inoculated leaves of Col-0 plants infected with each mutant virus was performed as described above to verify the presence of the introduced mutations and the absence of additional mutations.

### Characterization of virus infection

Upper non-inoculated leaves of the inoculated plants were collected and pooled from 16 plants at 14 days post-inoculation (dpi) for the extraction of total RNAs and proteins, respectively, as described (65). Total RNAs (5 and 20 μg) were loaded for Northern blot detection of the viral genomic/subgenomic RNAs and small RNAs by α-^32^P- and γ-^32^P-labeled probes, respectively (82). Hybridization signals were scanned by Amersham Typhoon scanner (Cytiva) and the intensity for the viral RNA 3 analyzed by ImageQuant TL v10.2 (Cytiva). For Western blotting, total proteins were separated into 12% polyacrylamide gel electrophoresis and transferred to a 0.45-μm nitrocellulose membrane (GVS North America) before the detection of the viral coat protein (CP) and 2b protein using the mouse anti-Fny-CMV CP antibody (1:5,000 dilution) and the rabbit anti-Fny-2b protein (1:1,000 dilution), respectively (23, 83). Detection of tubulin on the same membrane was done, with mouse anti-tubulin alpha chain antibody (Agrisera, AS20 4483, 1:5,000 dilution) serving as the loading control. Secondary antibodies used for the detection were goat anti-mouse IgG-HRP (Invitrogen, G-21040, 1:5,000 dilution) or goat anti-rabbit IgG-HRP (Thermo, 31460, 1:5,000 dilution). All experiments were repeated at least three times.

### Characterization of protein interactions

For yeast two-hybrid assay (Y2H), Cmv2b and 2b_1-93_ coding sequences were amplified with primer pairs BD-2bFp and BD-2bRp and BD-2bFp and BD-2b_1-93_Rp (Table S1), respectively, and cloned into pGBKT7 by infusion technology (Tolobio, 24305) to generate bait vectors pGBKT7-2b and pGBKT7-2b_1-93_. *Arabidopsis* RDR1 was cloned into pGADT7 yielding prey vector pGADT7-RDR1 with primer pairs AD-RDR1Fp and AD-RDR1Rp (Table S1). The bait and prey vectors were co-transformed into yeast strain Y2H gold cells according to the manufacturer’s instructions. Positive single colonies grown on the culture medium lacking synthetic dextrose/-Leu-Trp were subcultured, resuspended, and 10-fold serially diluted with sterile distilled water and then grown on the synthetic dextrose/-Ade-Leu-Trp-His culture medium at 30°C to detect protein interaction.

For co-immunoprecipitation (Co-IP) assay, Cmv2b and 2b_1-93_ coding sequences were amplified with primer pairs 2b-GFPFp/2b-GFPRp and 2b-GFPFp/2b_1-93_-GFPRp, respectively (Table S1), and cloned into the binary expression vector to generate GFP fusion vectors. *Arabidopsis* RDR1 was amplified with primers RDR1-FLAGFp and RDR1-FLAGRp (Table S1) and cloned into the binary expression vector, generating the FLAG fusion vector. Protein infusion vectors were transformed into *Agrobacterium tumefaciens* strain GV3101 for co-expression in *N. benthamiana* leaves by infiltration. Leaves were collected at 48 h post-*Agrobacterium* infiltration and subjected to total protein extraction with lysis buffer containing 20 mM Tris-HCl (pH 7.5), 100 mM NaCl, 2 mM DTT, 1 mM EDTA, 0.5% Triton X-100, and protease inhibitor. Fifty microliters of the total protein was kept as input control samples, and the remaining total protein was immunoprecipitated at 4°C for 3 h with anti-FLAG agarose beads (AlpalifeBio, KTSM1308). The agarose beads were then collected and washed three times with washing buffer containing 20 mM Tris-HCl (pH 7.5), 100 mM NaCl, 1 mM EDTA, and 0.1% Triton X-100. The bound proteins were eluted from agarose beads and subjected to Western blot detection with anti-FLAG (Abmart, M20008, 1:5,000 dilution) and anti-GFP (Abmart, M20004, 1:5,000 dilution) antibodies.

### Statistical analysis

Statistical analyses were performed on the data of the intensity of Northern blotting from four independent experiments. Normality and homoscedasticity tests were performed to determine the significance of the differences between the control and each experimental group by one-sided unpaired Student’s *t* test with Welch’s correction, without assuming equal standard deviations, using GraphPad Prism 9, with a *P* value less than 0.05 considered statistically significant.

## SUPPLEMENTAL MATERIAL

Table S1 mbio.01237-26-s0001.docx Sequences and usage of primer pairs.

ASM does not own the copyrights to Supplemental Material that may be linked to, or accessed through, an article. The authors have granted ASM a non-exclusive, world-wide license to publish the Supplemental Material files. Please contact the corresponding author directly for reuse.
