# Supplementary material for: A small viral protein suppresses immune amplification by two distinct mechanisms
Source: mBio. 2026 Jul 29;17(9):e01237-26. doi: 10.1128/mbio.01237-26 (PMC13556229; doi:10.1128/mbio.01237-26)
Supplement: Supplementary file 1 [file mbio.01237-26-s0001.docx]

**SUPPLEMENTAL MATERIAL**

**Table S1. Sequence and usage of primer pairs.**

| **Name** | **Sequence (5’ to 3’)** | **Usage** |
| --- | --- | --- |
| CMV-2aTFp | GCGATAGTGATATGGAATTGAACG | Generating pGEM-2aT and pGEM-2aT2b_1-93_ |
| CMV-2aTRp | TTCCATATCACTATCGCTGTTTGT |  |
| CMV-2b_18-110_Fp | GCTCGTATGGTGGAGGCGAAG | Generating pGEM-2b_18-110_ |
| CMV-2b_18-110_Rp | CTCCACCATACGAGCCAGTTG |  |
| CMV-2b_1-93_Fp | GATTAGTAGGATACAGATTGGTTCGCCGG | Generating pGEM-2b_1-93_ |
| CMV-2b_1-93_Rp | CCTACTAATCATGGTCTTCCGCCGATA |  |
| CMV-2b_1-76_Fp | GTGACCCGAGTCTGAG | Generating pGEM-2b_1-76_ |
| CMV-2b_1-76_Rp | CGAGATCACTCAGACTCGGGTC |  |
| CMV-RNA2Fp | CACTTCATGAGCTTTGTTGATCG | Amplify 2b region for sequencing |
| CMV-RNA2Rp | GGATGGACAACCCGTTCACC |  |
| BD-2bFp | TGCATATGGCCATGGAGGCCATGGAATTGAACGTAGGTGC | Generating Cmv2b bait vector |
| BD-2bRp | TGCGGCCGCTGCAGGTCGACTCAGAAAGCACCTTCCGCCCA |  |
| BD-2b_1-93_Rp | TGCGGCCGCTGCAGGTCGACTCAATCATGGTCTTCCGCCGATA | Generating 2b_1-93_ bait vector |
| AD-RDR1Fp | ATATGGCCATGGAGGCCAGTATGGGGAAGACAATTCAAGTG | Generating RDR1 prey vector |
| AD-RDR1Rp | ATCTGCAGCTCGAGCTCGATTCAACCGAAACGCAGAACATGG |  |
| 2b-GFPFp | GCATCTAGAAGGCCTACTAGTATGGAATTGAACGTAGGTGC | Generating Cmv2b-GFP fusion protein |
| 2b-GFPRp | CCCGGGGGTACCGTCGACGAAAGCACCTTCCGCCCATTC |  |
| 2b_1-93_-GFPRp | CCCGGGGGTACCGTCGACTCAATCATGGTCTTCCGCCGATA | Generating Cmv2b_1-93_-GFP fusion protein |
| RDR1-FLAGFp | GCATCTAGAAGGCCTACTAGTATGGGGAAGACAATTCAAGTG | Generating RDR1-FLAG fusion protein |
| RDR1-FLAGRp | CCCGGGGGTACCGTCGACACCGAAACGCAGAACATGGTC |  |
